# Impact of COVID-19 on Respiratory Admissions in a Tertiary Pediatric Intensive Care Unit

**DOI:** 10.7759/cureus.25369

**Published:** 2022-05-26

**Authors:** Shashikanth Ambati, Maya Mihic, Kristy Wilkinson, Javier L Sanchez, Chad Pezzano

**Affiliations:** 1 Division of Pediatric Critical Care, Albany Medical Center, Albany, USA; 2 Department of Pediatrics, Albany Medical Center, Albany, USA; 3 Department of Respiratory Medicine, Albany Medical Center, Albany, USA

**Keywords:** children, respiratory diseases, viral respiratory illness, covid-19, pediatric intensive care unit

## Abstract

Background

Pediatric inpatient admissions for viral respiratory infections decreased worldwide during the early part of the coronavirus disease 2019 (COVID-19) pandemic. This was likely due to social distancing measures and mask mandates leading to a decreased spread of viruses. We question if there was an increase in respiratory admissions during the winter of 2020-2021 due to the overlap of seasonal respiratory viruses and COVID-19 and the severity of those admissions.

Methods

We performed a single-center retrospective chart review of all respiratory admissions to our pediatric intensive care unit (PICU) from October to April during the years 2018-2019, 2019-2020, and 2020-2021. We compared the total number of respiratory admissions from different viruses and respiratory admissions by diagnoses among those time periods. Second, we compared the PICU length of stay and duration of mechanical ventilation (both invasive and non-invasive) for these respiratory admissions during those years.

Results

We saw a drastic decrease in the total respiratory admissions to the PICU in 2020-2021 compared to the same period of time in the last two years. The greatest contributor to this decrease was admissions secondary to bronchiolitis. We noticed a statistically significant decrease in both asthma (p<0.001) and chronic respiratory failure admissions (p=0.0029) during the pandemic winter compared to previous winters. Although, the total number of all respiratory viral admissions is not significant, admissions specific to the respiratory syncytial virus (RSV) (p<0.0001), rhino-enterovirus (p<0.0001), and multi-virus (p=0.0016), achieved statistical significance. There was no statistical difference between the PICU length of stay and duration of mechanical ventilation during the three years.

Conclusion

Despite a decrease in pediatric respiratory admissions during the COVID-19 pandemic, the severity of illness based on length of stay in the PICU and length of time on respiratory support remains unchanged compared to the previous two years.

## Introduction

The novel coronavirus disease 2019 (COVID-19) pandemic has had great effects on health systems worldwide. It not only crowded the intensive care units with COVID-19 admissions but also affected patients with other pathologies [1]. Adult hospitals in the United States have seen a reduction in the number of admissions due to non-COVID-19 reasons during this pandemic [2]. Children have been largely spared from the direct impact of the pandemic. There was only a small percentage of children and neonates affected with severe acute respiratory syndrome coronavirus-2 (SARS-CoV-2) infection or the associated hyper-inflammatory syndrome for whom admission to a pediatric intensive care unit (PICU) was required [3-4]. While the major impact of the COVID-19 pandemic has been on adult intensive care units, there has been some indirect impact on the pediatric health system. Children’s hospitals throughout the country have seen a reduction in pediatric admissions after the implementation of social distancing and masking requirements compared to the same periods in previous years [5].

Generally, the winter months from November through March are the busiest time in the intensive care units throughout the country due to respiratory viral diseases, asthma exacerbations, and bacterial infections such as pneumonia [6]. Though children were largely spared from SARS-CoV-2 infection from April to October 2020 [7], there were concerns for an increased burden on children’s hospitals during the winter months due to the overlap of COVID-19 and seasonal respiratory viruses. It is unknown, however, whether strategies implemented to mitigate COVID-19 spread could influence the epidemiology of concurrent seasonal viral lower respiratory tract infections (LRTIs) in children. We sought to study the impact of the COVID-19 pandemic on the number of PICU admissions for respiratory causes such as viral bronchiolitis, asthma exacerbations, and pneumonia. Second, we also postulate that the duration of the respiratory support and PICU hospitalization for those respiratory illnesses during the pandemic will be shorter compared to the previous year’s winters assuming less severity of the pathogens due to hygiene measures such as hand-washing and mask-wearing.

## Materials and methods

This is a single-center, retrospective chart review of all children who were admitted to our tertiary PICU at Albany Medical Center (AMC). Children between the ages of one month to 21 years of age who were admitted or discharged from our PICU with a respiratory diagnosis during the years 2018-2021 were included. The above information was obtained from our AMC PICU database. Our PICU at AMC maintains a separate database, which includes the date of admission, time of admission to the PICU, date of discharge or transfer, time of discharge from the PICU, and diagnosis of the patient. Respiratory diagnoses, such as bronchiolitis, asthma, croup, pneumonia, chronic respiratory failure, cystic fibrosis, and upper respiratory infections, were all included.

Inclusion criteria included children between the ages of one month and 21 years of age, admission to PICU during the months of October to April for the years 2018-2019, 2019-2020, and 2020-2021, and respiratory diagnoses as mentioned above. Exclusion criteria included non-respiratory conditions admitted to PICU. Once we derived the list of all the patients with respiratory diagnoses that were admitted to our PICU during the study period, then we used our electronic medical system to obtain the details of each patient. Data collection included different types of viruses such as the respiratory syncytial virus (RSV), rhino-enterovirus, influenza, human metapneumovirus (HMNV), adenovirus, coronavirus non-SARS, and multiple viruses (more than one virus), age of the patient, duration of PICU stay, type of respiratory support needed, duration of mechanical ventilation (invasive and non-invasive), and comorbidities such as bronchopulmonary dysplasia, prematurity, congenital heart disease, neuromuscular disease, sickle cell disease, immunocompromised, developmental delay, cerebral palsy, and Down syndrome.

We compared the total number of all respiratory admissions from different viruses and all respiratory admissions by diagnoses in the above-mentioned time periods. Second, we compared the PICU length of stay and duration of mechanical ventilation (both invasive and non-invasive) for these respiratory admissions during those years. The study was approved by the Albany Medical Center Institutional Review Board and informed consent was waived.

We used the chi-square test to compare the respiratory admissions by virus type and diagnoses among the three time periods, and reported p-values. Age, PICU length of stay, and length of respiratory support were reported using means and standard deviations, and the Kruskal-Wallis test was used to analyze that data. A p-value of less than 0.05 was used as a point of statistical significance. GraphPad Prism version 5.04 (GraphPad Software, San Diego, CA) was used for statistical analysis.

## Results

We saw a substantial decrease in respiratory admissions in the year 2020-2021 compared to the last two respiratory seasons. The greatest contribution to this decrease was a reduction in bronchiolitis admissions, although no statistically significant difference was noted (p=0.19). There was a decrease in both asthma and chronic respiratory failure admissions during the winter of 2020-2021, though they were both statistically significant (p<0.001 and p=0.0029, respectively) as described in Table 1.

**Table 1:** Total admissions by diagnosis SARS: Severe Acute Respiratory Syndrome

	2018 – 2019	2019 – 2020	2020 – 2021	p
Total admissions by diagnosis (n)	193	254	65	
Total admissions by virus type (n)	156	225	39	
Respiratory syncytial virus	71	59	1	p=<0.0001
Rhino-Enterovirus	34	78	37	p=<0.0001
Influenza	7	16	0	p=0.0697
Human metapneumovirus	7	16	0	p=0.0697
Adenovirus	0	2	0	p=0.3607
Corona non-SARS	4	3	0	p=0.4321
Multiple viruses	33	51	1	p=0.0016
Bronchiolitis admissions (n)	106	145	29	p=0.1964
Respiratory syncytial virus	60	53	1	p=0.0008
Rhino-Enterovirus	6	38	22	p=0.0001
Influenza	1	5	0	p=0.2951
Human metapneumovirus	5	3	0	p=0.368
Adenovirus	0	0	0	NS
Coronavirus non-SARS	1	2	0	p=0.4533
Multiple viruses	26	39	1	p=0.0677
Other	7	5	5	p=0.0170
Asthma admissions (n)	32	53	19	p = < 0.001
Croup admissions (n)	9	8	1	p = 0.4732
Pneumonia admissions (n)	13	21	7	p = 0.6238
Chronic respiratory failure admissions (n)	23	9	8	p = 0.0029
Cystic fibrosis admissions (n)	1	2	0	p = 0.7518
Upper respiratory infection admissions (n)	9	16	1	p = 0.3098

Along with a decrease in total admissions by all viral types in 2020-2021, we observed a statistically significant decrease in patients testing positive for RSV (p<0.0001). This observation correlates with the reduction in bronchiolitis admissions. There was also a decrease in patients testing positive for the rhino-enterovirus or multiple viruses upon admission in 2020-2021 as compared to previous years with statistically significant differences (p<0.0001 and p=0.0016, respectively). It should also be noted that this year, there were no admissions positive for influenza, HMNV, adenovirus, or corona non-SARS, which is atypical for the respiratory season. Information about viral admissions is also depicted in Table 1.

When comparing mean age, length of stay, and length of time on respiratory support based on diagnosis, we saw no statistically significant difference among the three years in either bronchiolitis, asthma, pneumonia, or chronic respiratory failure. The only trend noted was the increasing patient age in bronchiolitis admissions over the study period. Data on age, length of stay, and length of time on respiratory support for each diagnosis is depicted in Tables 2-5.

**Table 2:** Bronchiolitis admissions

Mean ± Standard Deviation	2018 – 2019	2019 – 2020	2020 – 2021	p
Mean Age (months)	9.68 ± 9.12	10.25 ± 9	15.9 ± 9.9	p = 0.3679
Length of Stay	3.76 ± 2.52	4.99 ± 6.79	2.2 ± 0.89	p = 0.3679
Length of Respiratory Support	3.41 ± 2.35	5.715 ± 8.32	2.1 ± 1.2	p = 0.3679

**Table 3:** Pneumonia admissions

Mean ± Standard Deviation	2018 – 2019	2019 – 2020	2020 – 2021	p
Mean Age (years)	9.53 ± 6.44	9.75 ± 5	10.6 ± 6.4	p = 0.3679
PICU Length of Stay	6.53 ± 9.84	6.0 ± 4.4	7.0 ± 6	p = 0.3679
Length of Respiratory Support	7.38± 9.6	6.24 ± 4.98	7.0 ± 5.9	p = 0.3679

**Table 4:** Asthma admissions

Mean ± Standard Deviation	2018 – 2019	2019 – 2020	2020 – 2021	p
Mean Age (years)	7.39 ± 4.54	6.4 ± 4.5	7.9 ± 4.7	p = 0.3679
PICU Length of Stay	2.25 ± 2.26	2.66 ± 1.5	2.5 ± 1.7	p = 0.3679
Length of Respiratory Support	2.5 ± 2.41	2.6 ± 1.6	2.4 ± 1.5	p = 0.3679

**Table 5:** Chronic respiratory failure admissions

Mean ± Standard Deviation	2018 – 2019	2019 – 2020	2020 – 2021	p
Mean Age (years)	5.91 ± 4.98	10.04 ± 5.77	6.4 ± 5.9	p = 0.3679
PICU Length of Stay	9.17 ± 13	4.78 ± 2.77	5.6 ± 6.2	p = 0.3679
Length of Respiratory Support	12.34 ± 17.4	4.82 ± 3.18	5.7 ± 6.2	p = 0.3679

## Discussion

In the United States, respiratory illnesses are the most common reason for PICU admissions in children [8]. COVID-19 public health interventions have impacted the admissions in pediatric ICUs throughout the country. The majority of children in the United States who have been infected with the SARS-CoV-2 virus only experienced a mild illness that did not require hospitalization [9]. Generally, viral lower respiratory tract infections have a seasonal pattern, resulting in many PICU admissions worldwide, primarily during the winter months [10]. In late summer 2020, there was concern among the intensivists that there would be an increased burden on the children’s hospitals during the winter months due to the overlap of COVID-19 and seasonal viruses. On the contrary, we have seen drastically reduced respiratory admissions during the period from October 2020 to April 2021 as described in Table 1. Although the numbers of all respiratory tract infections during the study period were lower than in previous years, these results were not statistically significant, except for asthma and children with chronic respiratory failure. Our results correlate with other studies in the country, which showed a similar reduction in respiratory admissions to children’s hospitals during the months of October to April 2020-2021 [11-13]. Similar results were published in other countries, such as Brazil and Italy, in time periods that are not typical for these respiratory admissions [2,14]. To date, our study is the only one that has examined changes in the use of PICU due to seasonal viral LRTIs during the winter season of the COVID-19 pandemic.

Bronchiolitis and asthma are the leading causes of hospitalization among children [15-16]. These conditions are triggered by a variety of viruses such as RSV, rhino-enterovirus, influenza, coronavirus non-SARS, HMNV, and adenovirus. The recent introduction and widespread use of molecular-based methods (real-time multiplex polymerase chain reaction (PCR)), has helped identify an increased number of concomitant viral infections in children [17]. In this single-center, retrospective study, we found a significant and substantial decrease in the total number of RSV, rhino-enterovirus, and multi-viral admissions in the winter during the COVID-19 pandemic as compared to previous years (Table 1). Although there were no cases of influenza, human metapneumovirus, adenovirus, and coronavirus during the study period, the results did not reach significance. We believe that the associated public health measures enacted to slow the spread of COVID-19 such as mask-wearing, hand-washing, and social distancing may have affected the transmission of these other respiratory pathogens, resulting in decreased numbers. The marked reduction in critical asthma admissions suggests that infections contribute substantially to asthma exacerbations although reduced allergen exposure due to mask-wearing and increased parental supervision may also have influenced this finding.

We hypothesized that the severity of respiratory tract infections caused by these viruses would be decreased secondary to the predictable effects of mask mandates and social distancing with resultant lower viral loads. We expected to have these children to have a shorter duration of respiratory support and decreased length of stay. Interestingly, none of the respiratory tract infections have achieved statistical significance for both variables indicating the severity of these viruses is not different despite public health measures.

Children with chronic medical problems, including those with chronic respiratory failure who are ventilator-dependent, congenital heart disease, prematurity, and bronchopulmonary dysplasia, and neuromuscular diseases are presumed to be medically vulnerable to both risk of viral infection and more severe disease [18]. We looked at our cohort of patients to see if there was a higher proportion of patients admitted with co-morbidities in 2020-2021 compared to the previous two years to explain the reason for viral admissions this year despite social distancing measures. The proportion of patients with a respiratory illness that had comorbidities in 2018-2019 was 47%, 2019-2020 was 46%, and in 2020-2021 was 42%. There was a high incidence of patients admitted with co-morbidities during all three time periods with no huge increase in the most recent year.

There was a significant decrease in emergency department visits and hospitalizations among children during the pandemic [19]. One large retrospective study recently published that during the pandemic, admissions to children’s hospitals for all causes had decreased, with the greatest impact being from a decrease in respiratory illness [20]. These declines have been attributed to various health measures such as wearing face masks, hand hygiene, social distancing, and school closing [21-22]. Interestingly, this reduction was observed while daycare remained open, and schools reopened in September 2020. Nonetheless, it is also plausible that school attendance may have decreased due to hybrid in-person/remote learning or that infection control measures instituted in schools may have prevented viral transmission [23].

Our study is limited by being a single-center observational cohort with viral respiratory tract infections conducted through retrospective analysis. Although the total number of admissions caused by RSV, rhino-enterovirus, and multiple viruses were the only numbers that achieved statistical significance along with asthma and chronic respiratory failure admissions, we believe that a multicentered study with diverse characteristics from every region of the country could yield results with statistical significance.

## Conclusions

There had been a major concern among pediatricians during the spring of 2020 for a perfect storm in winter where SARS-CoV-2 converges with seasonal respiratory viruses, resulting in a higher burden on children’s hospitals, with disastrous consequences. On the contrary, we have seen a drastically reduced number of admissions due to respiratory illnesses during the winter of 2020, with a reduction in asthma admissions reaching statistical significance. Overall, the number of RSV and rhino-enterovirus admissions have significantly decreased, with no recorded admissions positive for influenza, human metapneumovirus, and adenovirus. Public health intervention measures, such as mask mandates, hand hygiene measures, social distancing, and school closures during the early pandemic, could have led to decreased transmission of these pathogens, but they remained decreased despite daycares remaining open and schools reopening with hybrid learning.
